# Confirmation of *TACO1* as a Leigh Syndrome Disease Gene in Two Additional Families

**DOI:** 10.3233/JND-200510

**Published:** 2020-06-02

**Authors:** Yavuz Oktay, Serdal Güngör, Lena Zeltner, Sarah Wiethoff, Ludger Schöls, Ece Sonmezler, Elmasnur Yilmaz, Benjamin Munro, Benjamin Bender, Christoph Kernstock, Sofie Kaemereit, Inga Liepelt, Ana Töpf, Uluc Yis, Steven Laurie, Ahmet Yaramis, Stephan Zuchner, Semra Hiz, Hanns Lochmüller, Rebecca Schüle, Rita Horvath

**Affiliations:** a Izmir Biomedicine and Genome Center, Dokuz Eylul University Health Campus, Izmir, Turkey; b Izmir International Biomedicine and Genome Institute, Dokuz Eylul University, Izmir, Turkey; c Inonu University, Faculty of Medicine, Turgut Ozal Research Center, Department of Paediatric Neurology, Malatya, Turkey; d Department of Neurodegenerative Diseases, Hertie-Institute for Clinical Brain Research and Center of Neurology, University of Tübingen, Tübingen, Germany; e German Center for Neurodegenerative Diseases (DZNE), University of Tübingen, Tübingen, Germany; f Department of Clinical Neurosciences, University of Cambridge School of Clinical Medicine, Cambridge Biomedical Campus, Cambridge, UK; g Diagnostic and Interventional Neuroradiology, Radiologic Clinics, University of Tübingen, Tübingen, Germany; h Centre for Ophthalmology, University Eye Hospital Tübingen, University of Tübingen, Tübingen, Germany; i John Waltom Muscular Dystrophy Research Centre, Translational and Clinical Research Institute, Newcastle University, Newcastle upon Tyne, UK; j Dokuz Eylul University, School of Medicine, Department of Paediatric Neurology, Izmir, Turkey; k CNAG-CRG, Centre for Genomic Regulation, Barcelona Institute of Science and Technology, Barcelona, Spain; l Pediatric Neurology Clinic, Private Office, Diyarbakir, Turkey; m Department of Human Genetics and Hussman Institute for Human Genomics, University of Miami Miller School of Medicine, Miami, FL, USA; n Department of Neuropediatrics and Muscle Disorders, Medical Center–University of Freiburg, Faculty of Medicine, Freiburg, Germany; o Children’s Hospital of Eastern Ontario Research Institute; Division of Neurology, Department of Medicine, The Ottawa Hospital; and Brain and Mind Research Institute, University of Ottawa, Canada

## Abstract

**Background:**

In 2009, we identified *TACO1* as a novel mitochondrial disease gene in a single family, however no second family has been described to confirm the role of *TACO1* in mitochondrial disease.

**Objective:**

In this report, we describe two independent consanguineous families carrying pathogenic variants in *TACO1*, confirming the phenotype.

**Methods:**

Detailed clinical investigations and whole exome sequencing with haplotype analysis have been performed in several members of the two reported families.

**Results:**

Clinical phenotype of the patients confirms the originally reported phenotype of a childhood-onset progressive cerebellar and pyramidal syndrome with optic atrophy and learning difficulties. Brain MRI showed periventricular white matter lesions with multiple cystic defects, suggesting leukoencephalopathy in both patients. One patient carried the previously described homozygous *TACO1* variant (p.His158ProfsTer8) and haplotype analysis suggested that this variant is a rare founder mutation. The second patient from another family carried a homozygous novel frame shift variant (p.Cys85PhefsTer15).

**Conclusions:**

The identification of two Turkish families with similar characteristic clinical presentation and an additional homozygous nonsense mutation confirms that *TACO1* is a human mitochondrial disease gene. Although most patients with this clinical presentation undergo next generation sequencing analysis, screening for selected founder mutations in the Turkish population based on the precise clinical presentation may reduce time and cost of finding the genetic diagnosis even in the era of massively parallel sequencing.

## INTRODUCTION

Next generation sequencing (NGS) has led to the discovery of a large number of human disease genes in rare inherited diseases [1] however, due to the rarity of some gene defects, the confirmation of the gene-phenotype association by identifying different mutations in independent families could not have been confirmed for all genes. In 2009, we identified a homozygous frame shift variant in the first specific mitochondrial translational activator, *TACO1* [2] in 5 patients of a consanguineous Kurdish family with juvenile-onset Leigh-like syndrome, short stature, optic atrophy, dystonia, spastic tetraparesis, dysarthria and cognitive impairment [3].This was the first example, when mutations in a nuclear gene affect the translation of a single mtDNA-encoded protein. Impaired mitochondrial transcription and translation are responsible for >50% of mitochondrial disorders [4], but mutations in translational activators are still a very rare disease cause. No second family has been described in detail with *TACO1* mutations following the first description a decade ago.

*Taco1* mutant mice homozygous for a missense point mutation (c.491T>A, p.Ile164Asn) showed late-onset visual impairment, motor dysfunction and heart hypertrophy [5]. The mutation resulted in defective binding of TACO1 to multiple adenine-guanine rich sequences of mitochondrial cytochrome *c* oxidase subunit I (*MTCOI*) mRNA and its association with the mitochondrial ribosomes.

In this report, we describe two independent consanguineous families carrying pathogenic variants in *TACO1*, confirming the phenotype. To our knowledge, this is the first independent confirmation of *TACO1* – associated Leigh syndrome after the initial report by Weraarpachai et al. [2].

## PATIENTS AND METHODS

### Case presentations

#### Family 1

An 18-year-old male was born to consanguineous parents (first-grade cousins) after an uneventful pregnancy by caesarean section (2900 gr birth weight). He had normal early development until 4 years of age. His first symptoms included falls at age 4 years, which became more frequent by 6 years of age and he developed difficulty in climbing stairs. At age 7 years, he progressively lost motor skills. Vision loss and cognitive decline started at around age 8 years.

**Fig. 1:**
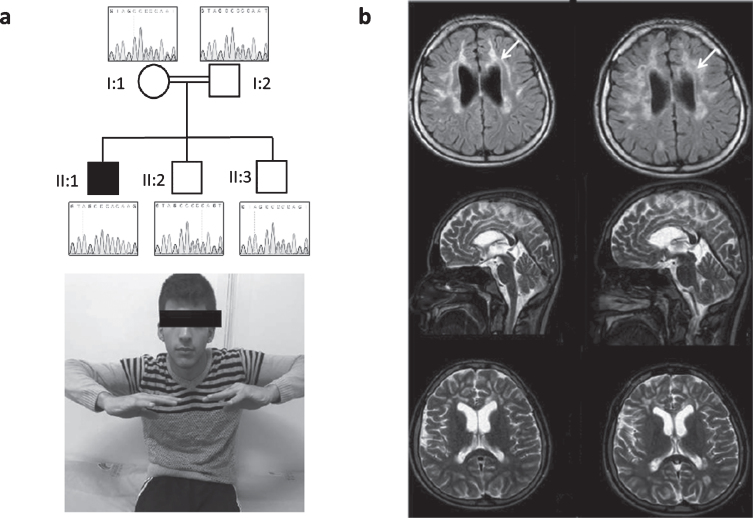
Patient 1, homozygous for c.472insC (p.His158ProfsTer8). (a) Neurological examination of the patient shows bilateral optic atrophy, upper extremity amyotrophy, proximal and distal weakness and spastic tetraparesis. Pedigree of the family and Sanger sequencing show segregation of the TACO1 variant in family members. The proband is homozygous and his mother, father and unaffected siblings are heterozygous for the *TACO1*; c.472insC variant. (b) Cranial MR imaging demonstrate progressive cerebral atrophy and T2 hyperintense periventricular white matter lesions (arrows).

Neurological examination showed bilateral optic atrophy, swallowing problems and dysarthria. He had proximal and distal weakness with upper limb amyotrophy, foot deformity and spastic tetraparesis with increased deep tendon reflexes and positive Babinski sign bilaterally. Coordination was impaired, he had limb ataxia and difficulty in walking. He showed urinary and bowel incontinence. He had learning difficulties with dysarthria, delayed speech and language development (Fig. 1).

Electromyographic findings showed myogenic changes and muscle biopsy revealed cytochrome *c* oxidase (COX) negative fibers. Laboratory blood results (including CK, ALT, AST, lactic acid) were all normal. Metabolic examinations showed normal organic acids, amino acids and lysosomal enzymes. Cranial MR revealed progressive cerebral atrophy and profound T2 hyperintense periventricular white matter lesions. Additionally, abnormalities of visual and auditory evoked potentials were recorded.

#### Family 2

The patient is a 39-year-old female, born after an uneventful pregnancy without complications to consanguineous parents, both originating from the same small village in Turkey. Her psychomotor development was delayed and she tended to walk on tiptoes. From age 4 years her spastic gait disturbance worsened significantly. At 15 years of age she was still able to walk independently for few meters while requiring a wheelchair to navigate longer distances. At age 27 years her ability to walk was reduced to <10 m with walking aids, around age 30 years she became wheelchair-bound. Her cognitive development was also delayed with delayed speech and language development and a learning disability; after leaving school at 20 years of age she started working at a sheltered workplace.

**Fig. 2:**
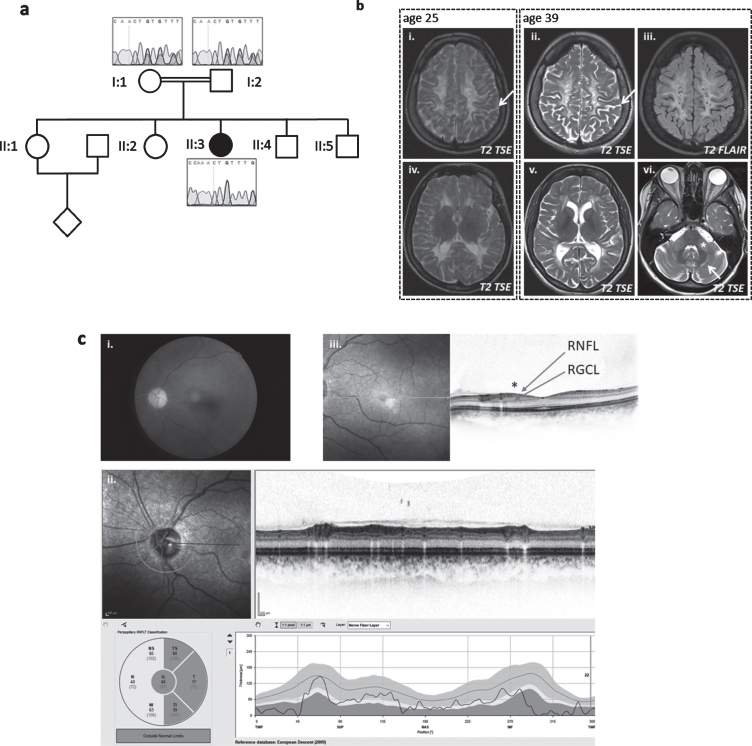
Patient 2, homozygous for c.252_253delCT (p.Cys85PhefsTer15). (a) Pedigree of family and Sanger sequencing show that the prob and is homozygous and her mother and father are heterozygous for the *TACO1*c.252_253delCT variant. (b) Baseline MRI image at the age of 25 years (*i* + iv on the left) and follow-up 14 years later (ii/iii+v/vi) show white matter degeneration with white matter atrophy and sulcal widening (arrows in i and ii) as well as cystic lesions that demonstrate CSF like signal behavior in the FLAIR (iii). The infratentorial T2-weighted image (vi)demonstrates hyperintensities within the middle cerebellar peduncle (asterix) and within the cerbellar white matter (arrow). (c) Color fundus photography (i) shows severe paleness of the optic nerve head. OCT (ii+iii) reveals atrophy of the peripapillary retinal nerve fiber layer (RNFL) (ii). The foveal depression is flattened due to atrophy of the ganglion cell layer (GCL) and retinal nerve fiber layer (RNFL) (see * in (iii)). All other retinal layers are intact; in particular, there are no signs or chorioretinal atrophy. Best corrected visual acuity was 20/400 (right eye) and 20/250 (left eye), the subject was legally blind (according to WHO definition). Images for the left eye are shown; all changes were present in both eyes.

Neurological examination at age 39 years showed severely reduced visual acuity, dysarthria, cerebellar ataxia with saccadic pursuits and upper limb ataxia with intention tremor. She had brisk upper limb reflexes, pyramidal signs and reduced fine motor skills of the upper limbs and severe spastic paraplegia of the lower limbs with adductor and knee extensor spasticity (° 3–4 on the Ashworth scale), generalized lower limb weakness (∼3/5 on the MRC scale), brisk reflexes and extensor plantar sign. Ophthalmological examination confirmed an optic atrophy, progressive over time; no retinal changes were noted on fundoscopy (Fig. 2). Motor evoked potentials confirmed affection of the corticospinal tracts to upper and lower limbs. Visually evoked potentials demonstrated prolonged P100 latency. Nerve conduction studies were reported normal at age 28 years but indicated a sensorimotor peripheral neuropathy later in the disease course (first noted at age 34, progressive at age 39). Electromyography of the extensor carpi radialis muscle revealed small, short and polyphasic potentials with early recruitment and a full interference pattern, indicating myopathic changes (age 27 years).

Cranial MRI showed slowly progressive confluent T2 hyperintense signal changes of the periventricular and cerebellar white matter and middle cerebellar peduncles with multiple cystic defects, internal cerebral atrophy with dilatation of the lateral ventricles and thin corpus callosum. In the white matter, several areas with disturbed diffusion were noted, only some of which corresponded to areas of ADC reduction. No morphological or signal changes were noted in the area of the basal ganglia (Fig. 2). MRI spectroscopy revealed a strong lactate peak, indicating mitochondrial disease.

The probands and unaffected family members were consented for research that was approved by Dokuz Eylul University, School of Medicine Institutional Review Board (family 1) and the University of Tübingen School of Medicine Institutional Review Board (family 2). Informed consent was obtained from all family members that were recruited to the study prior to participation including for whole exome sequencing and the publication of medical information.

### Whole exome sequencing and Sanger sequencing

DNA isolation from peripheral blood was done according to a standard protocol. Whole exome sequencing (WES) was performed on DNA obtained from index, unaffected siblings and parentsof family 1 at the Broad Institute of MIT and Harvard, using Illumina Exome Capture Kit (38 Mb target) and in family 2 at the Hussman Institute for Human Genomics (HIHG) at the University of Miami, using Agilent SureSelect Capture (v4, 51 Mb). Sequencing data was processed at the Centro Nacional de Análisis Genómico (CNAG), Barcelona, and data analysis carried out on the RD-Connect Genome-Phenome Analysis Platform (https://platform.rd-connect.eu/genomics) for family 1 and analyzed on the Genesis web-based analysis and collaboration platform (https://www.genesis-app.com) maintained by the Genesis Project Foundation (https://www.tgp-foundation.org) [6] for family 2 using standard filtering criteria for rare diseases, including Minor Allele Frequency (MAF) <0.01, Variant Effect Predictor (VEP) = moderate/high and Combined Annotation Dependent Depletion (CADD) >20.

### Haplotype analysis

We selected 18 SNPs in *TACO1* and in the surrounding genes in the proximity of the c.472insC, p.His158ProfsTer8 mutation. the Sequence information surrounding each of 18 SNPs of interest (and *TACO1*) was obtained via the USCS genome browser (https://genome-euro.ucsc.edu/index.html) (GRCh37/hg19 assembly) and primers to amplify these regions were designed using the Primer3web application (http://primer3.ut.ee/) (available on request). Purified PCR products were sequenced with an Applied Biosystems 3730xl DNA Analyser. Sequences were analysed using Chromas version 2.6.6.

## RESULTS

Whole exome sequencing (WES) in family 1 revealed the previously reported homozygous one base-pair insertion at codon 472 in the *TACO1* gene (c.472insC, p.His158ProfsTer8) in the index patient that causes a frame-shift and creates a premature stop codon leading to the classification pathogenic/damaging [1]. The variant was confirmed by Sanger sequencing and was homozygous in the patient and heterozygous in his parents and healthy siblings (Fig. 1).

In family 2, a homozygous 2-bp deletion (c.252_253delCT) was identified by WES and confirmed by Sanger sequencing. The change causes a frame-shift and introduces a premature stop codon (p.Cys85PhefsTer15). Both parents were heterozygous for the change (Fig. 2).

**Fig. 3:**
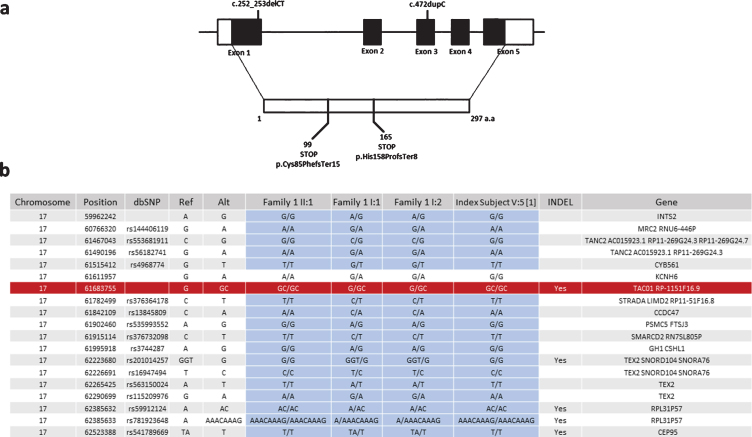
Schematic representation of exon-intron structure of *TACO1* and the mutations detected in our patients. (a) Schematic representation of exon-intron structure of the *TACO1* gene and the reported mutations c.472insC (p.His158ProfsTer8) andc.252_253delCT (p.Cys85PhefsTer15). (b) Haplotype analysis of the SNPs in the proximity of the c.472insC (p.His158ProfsTer8) mutation suggesting a founder effect for this variant.

As we identified the same c.472insC, p.His158ProfsTer8 variant in family 1, which we reported previously in another consanguineous Turkish family, we studied whether this mutation arose on the same haplotype. We identified 18 SNPs surrounding the variant on exome sequencing of family one and tested these in DNA of the index patient from the original publication in 2009. Sanger sequencing analysis showed that only one of the 18 variants was different in the original patient compared to family 1 in this study, suggesting a founder effect (Fig. 3).

## DISCUSSION

Cytochrome *c* oxidase (COX) or complex IV is the terminal electron acceptor in mitochondrial electron transport chain [7, 8]. It functions as a dimer and its monomer, with 13 subunits, is controlled by both the nuclear and mitochondrial genome. The three largest, catalytic subunits (COX I, II and III) are encoded by the mitochondrial DNA. The remaining ten structural subunits and many regulatory genes that have roles in assembly and biogenesis of the COX enzyme are encoded by nuclear DNA [7–9].

In 2010, Seeger et al. [3] described in detail the clinical presentation of the original and single consanguineous Kurdish family with 5 affected individuals homozygous for a frame-shift mutation that results in truncated TACO1 protein [2]. Here, we describe two additional families: one patient carried the previously described homozygous *TACO1* variant (p.His158ProfsTer8), and a second patient from another family carried a homozygous novel frame shift variant (p.Cys85PhefsTer15). Clinical phenotype of the patients confirms the originally reported phenotype of a childhood-onset progressive cerebellar and pyramidal syndrome with optic atrophy and learning difficulties. Hyperintense periventricular white matter lesions with multiple cystic defects, suggesting leukoencephalopathy were the most prominent MRI changes common in all patients, however thin corpus callosum, white matter changes in the cerebellum and brainstem and progressive cerebral atrophy were also reported in the MRI of some affected individuals. The clinical and MRI findings of our patients are strikingly similar to the originally reported phenotype.

There are no additional mutations reported in *TACO1* in the last 10 years, however a *Taco1*^mut/mut^ mouse model has been recently reported. The presentation of the *Taco1*^mut/mut^ mice with late-onset visual impairment, motor dysfunction is similar to the phenotype observed in patients [5], except for cardiac hypertrophy, which was detected in the *Taco1^*mut*/*mut*^* mice, but not present in the patients. This mouse model is useful to study the disease mechanism and may benefit future treatment studies in TACO1 deficiency.

Advances in NGS technologies led to the establishing of better clinical diagnosis and appropriate management of the patients with clinically heterogeneous mitochondrial diseases. The patients described in this study were initially suspected of having a leukodystrophy based on the severe white matter lesions on MRI or complicated Hereditary Spastic Paraplegia (HSP) based on the progressive spastic paraparesis and NGS resulted in the diagnosis of mitochondrial disease. Our findings provide support for the hypothesis that the incidence of mitochondrial etiology of neurogenetic disorders with white matter lesions is more common than expected.

Although we are not aware of a common ancestor relationship between family 1 reported here and the previously reported family, haplotype analysis suggested that this variant is a rare founder mutation. In summary, *TACO1* mutations present with a childhood-onset, slowly progressive spastic paraparesis, ataxia and optic neuropathy with characteristic cystic white matter lesions in the periventricular area and cerebellum. Screening for c.472insC variant in *TACO1* should be considered in patients, particularly from consanguineous Turkish marriages, even without data on muscle histology or respiratory chain deficiency. Although most patients with this clinical presentation undergo next generation sequencing analysis, screening for selected founder mutations in the Turkish population based on the precise clinical presentation may reduce time and cost of finding the genetic diagnosis even in the era of massively parallel sequencing.

## FUNDING

The study was supported by the Newton Fund UK/Turkey (MR/N027302/1 to HL and RH), the Medical Research Council (UK) (MR/N025431/1 to RH), the Wellcome Investigator fund (109915/Z/15/Z to RH), the Lily Foundation UK (to RH), the European Research Council (309548 to RH); and the Wellcome Trust Pathfinder Scheme (201064/Z/16/Z to HL and RH), the TUBITAK project 216S771 to SH and YO. YO is supported by Turkish Academy of Sciences (TUBA) Young Investigator Program (TUBA-GEBIP). The study was further supported by the Horizon 2020 research and innovation program via grant 779257 “Solve-RD” to RS, the “Bundesministerium für Bildung und Forschung” (BMBF) via funding for the translational research consortium TreatHSP (https://treatHSP.net) (01GM1905 to RS), the National Institute of Health (NIH/NINDS) (grant 5R01NS072248 to RS and SZ) and the HSP Selbsthilfegruppe e.V. (grant to RS).HL receives support from the Canadian Institutes of Health Research (Foundation Grant FDN-167281), the Canadian Institutes of Health Research and Muscular Dystrophy Canada (Network Catalyst Grant for NMD4C),the Canada Foundation for Innovation (CFI-JELF 38412), and the Canada Research Chairs program (Canada Research Chair in Neuromuscular Genomics and Health, 950-232279).We thank the Broad Institute for carrying out WES. The Broad Center for Mendelian Genomics (UM1 HG008900) is funded by the National Human Genome Research Institute with supplemental funding provided by the National Heart, Lung, and Blood Institute under the Trans-Omics for Precision Medicine (TOPMed) program and the National Eye Institute. Data was analysed using the RD-Connect Genome-Phenome Analysis platform developed under FP7/2007-2013 funded project (grant agreement n° 305444) and using the Genesis platform developed and maintained by the Genesis Foundation (https://www.tgp-foundation.org).Several authors of this publication are members of the European Reference Network for Rare Neurological Diseases - Project ID No 739510 (BB, LS, LZ, RS) and EURO-NMD (HL, RH). SW is supported by the Ministry of Science, Research and the Arts of Baden-Württemberg and the European Social Fund (ESF) of Baden-Württemberg (31-7635 41/67/1).

## CONFLICT OF INTEREST

The authors have no conflict of interest to report.
